# Practical Medicine

**Published:** 1878-05

**Authors:** 


### Practical Medicine.

Extra-Pericardial Adhesions.—Dr. O. von Widmann. (*Virchow's Archiv* for July, 1877.)

That systolic retraction of the præcordia is not a trustworthy sign of these adhesions is generally believed. The essential factor in the ætiology of this retraction is a change in the position of the heart, with or without adhesions. Inasmuch as the left to right diameter of the heart is shortened during systole, if, for any reason, the organ were so turned as to make this the antero-posterior diameter, there would be a tendency to a vacuum with each systole, and atmospheric pressure might cause the retraction.

Dr. Riegel has called attention (*Berlin. Klin. Wochenschrift*, Nov., 1877) to the fact that, while the apex beat is more decidedly revealed on a cardiogram during expiration than during inspiration, the converse has been observed in several cases of pericardial adhesions to the border of the lung; and he explains it by the inability of the latter to come forward during inspiration, while in expiration they retract upon the pericardium and so impede the heart.

Copaiba and Cubebs in Croup.—Dr. Oldoni. (*Annali Universali di Medicina.*)

Five cases were cured by the following treatment:

| | | | |
|---|---|---|---|
| ℞ | Bals. copaibæ - - - | ℥ss. | |
| | Pulv. acaciæ - - - - | ʒv. | |
| | Aq. menth. pip. - - - | ℥iii. | m. |

Dessertspoonful every two hours.

| | | | |
|---|---|---|---|
| ℞ | Cubebæ pulv. recent. - - - | ℥ss. | |
| | Syrupi (simp.) - - - | ℥x. | m. |

Tablespoonful every two hours alternately with the above. Diminish the dose for children under four years of age.

His cases were very severe in every respect.

Feigning Fever. Sellerbecker, (*Berlin. Klin. Wochenschrift.*)

A peculiar case of feigning in order to secure greater attention is here reported. The chief points are as follows:

A female patient under treatment for stenosis of the heart and ulcer of the stomach:

Temperature found to vary, without apparent cause, from 101 to 103°. Pulse 120 and respirations 20.

The rapidity of the pulse was easily explained, and the doctor, by means of rapid and deep respirations, elevated his own pulse from 75 to 130 per minute.

The elevation of temperature was more difficult of explanation but was evidently effected by deceit. To clear up the point, the thermometer which in the axilla registered 101.1 was placed in the rectum which gave a temperture of only 99.6.

The patient eventually confessed and explained her methods the rectum.

As soon as the nurse had placed the thermometer in the axilla, from pretext of cold she covered herself well, and then drawing the back part of her chemise forward in the axilla, and making of it a kind of sack in which to envelope the thermometer, she pressed it firmly between the arm and thorax. Then by rapid respiration she produced friction of the thermometer between the folds of her chemise. This she continued until she obtained the desired elevation.

Dr. Sellerbecker, in trying this himself, produced a registration of 114.4°. This effect could not, on account of evaporation, be produced when the thermometer was subject to friction by direct contact with the skin. But when the skin was very dry, in the course of three minutes a temperature of 107.4° was obtained.

It would seem from this circumstance that the temperature in the axilla, when the respirations are rapid and skin dry, should always be corroborated by the temperature in the mouth or rectum.

Resuscitation in Poisoning and Asphyxia.—R. Boehm, (Arch. f. Exp. Path. und Pharmacologie. Bd. VIII. p. 68–100).

An injection of 0.1 gramme potassic nitrate, pushed rapidly into the veins of a cat causes, within a few minutes, a sinking of blood pressure, until it becomes zero. Respiration ceases, tetanic spasms set in and death is inevitable. If now artificial respiration is resorted to within 3–4 minutes after the stoppage of breathing, and the expiration is aided by alternate compression of the chest, the mercury of the manometer in the artery can be made to rise 50 to 120 millimeters with each compression; thus an artificial circulation may be maintained. Sometimes 4 to 6 compressions suffice, in other cases it requires 4 to 36 minutes to start the action of the heart, and to keep it up persistantly. This result is due not only to the supply of oxygen thus furnished to the nerves of the heart, but also to a mechanical stimulation of the heart by the compression. Within a time varying from 40 seconds to 54 minutes, the cardiac action is followed by natural breathing.

In apparent death from chloroform, resuscitation too can be effected by means of artificial respiration in those cases only, in which the heart has not yet fully stopped beating. If the latter accident has occurred, compression of the thorax becomes necessary, and even this loses its utility 7 to 19 minutes after the stoppage of the circulation and 10 to 24 minutes after cessation of breathing.

Sudden deprivation of oxygen (rapid asphyxia) paralyzes the heart much more rapidly. Artificial respiration combined with methodic compression of the thorax must be commenced at the latest 1 to 1½ minutes after stoppage of the heart, in order to succeed as means of resuscitation.

Resuscitated animals show a number of "symptoms of resuscitation from apparent death." Boehm describes them as increased reflex irritability as in animals poisoned with strychnia, in co-ordinated movements, diminished sensibility, blindness remaining for two to three days, probably also loss of hearing, taste and smell, reduction of temperature and finally transitory glycosuria. In general, the animals resemble for a time dogs, whose cerebrum has been removed by the method of Goltz. Similarly as in deep narcrosis, the different organs do not all return to a state of activity at once. The heart first begins to act, next respiration returns, and finally the functions of the brain are re-established.

PATHOLOGY OF CONCUSSION.—M. Duret. (*Société de Biologie.*)

Mere oscillatory disturbances of the encephalon do not offer a satisfactory explanation of the profound symptoms supervening upon a blow upon the head; nor do such blows, when they produce concussion, leave sufficient structural change.

Duret has been able to reproduce *all* the symptoms of concussion, by increasing the tension of the cerebro-spinal fluid, by perforating the cranium, and injecting various fluids.

He cut away the cervical muscles, laid bare the occipito-atloidean membrane, and watched its respiratory pulsations, or recorded them by the graphic method. When he injected fluid, as above, this membrane became tense, and all the clinical manifestations of shock followed; he then punctured it, and allowed the fluid to escape, and all these evidences disappeared.

By such experiments, and by others equally ingenious, he was confirmed in the impression that it was to tension of the cerebro-spinal fluid, and not to changes of the cerebral pulp, that we should ascribe the phenomena of concussion.

